# CMR-Derived Strain and Torsion Reveal Subclinical Dysfunction in Hypertrophic Cardiomyopathy: A Prospective Case–Control Study

**DOI:** 10.3390/biomedicines13081986

**Published:** 2025-08-15

**Authors:** Alexandru Zlibut, Ioana Danuta Muresan, Michael Bietenbeck, Andrei Dan Radu, Lucia Agoston-Coldea

**Affiliations:** 1 Department of Internal Medicine, Iuliu Hatieganu University of Medicine and Pharmacy, 400012 Cluj-Napoca, Romania; 2 Division of Cardiovascular Imaging, Department of Cardiology I, University Hospital Muenster, 48149 Muenster, Germany; 3 Laboratory of Interventional Cardiology, Carol Davila Central Military Emergency University Hospital, 050474 Bucharest, Romania; 4 Department of Radiology, Affidea Hiperdia Diagnostic Imaging Center, 400012 Cluj-Napoca, Romania; 5 Department of Internal Medicine, Emergency County Hospital, 400012 Cluj-Napoca, Romania

**Keywords:** hypertrophic cardiomyopathy, cardiac magnetic resonance imaging, left ventricle global longitudinal strain, left ventricle global torsion, left ventricle peak-systolic torsion

## Abstract

**Background**: Hypertrophic cardiomyopathy (HCM) is frequently associated with preserved left ventricular ejection fraction (LVEF), yet subclinical myocardial dysfunction often escapes detection using conventional imaging. Cardiac magnetic resonance (CMR) with feature tracking (FT) enables precise assessment of myocardial deformation and mechanics. **Methods**: In this prospective case–control study, we evaluated 150 HCM patients and 100 age- and sex-matched healthy controls using standardized CMR protocols. Global longitudinal strain (GLS), circumferential strain (GCS), radial strain (GRS), and left ventricular (LV) torsion were quantified via FT-CMR. Myocardial fibrosis was assessed through late gadolinium enhancement (LGE), native T1 mapping, and extracellular volume (ECV). **Results**: HCM patients showed significantly impaired strain and torsion metrics compared with controls: GLS (−16% vs. −20%), GCS (−18% vs. −21%), GRS (29% vs. 38%), and global LV torsion (1.27°/cm vs. 1.95°/cm), all *p* < 0.001. These abnormalities were also observed in LGE-negative patients, suggesting early functional remodeling. Global LV torsion demonstrated the highest diagnostic performance for LGE detection (AUC = 0.995), surpassing those of GLS (0.877), native T1 (0.731), and ECV (0.657). A cut-off value of 0.7°/cm provided optimal sensitivity and specificity, and was associated with adverse prognosis in survival analysis. **Conclusions**: CMR-derived strain and torsion parameters detect early myocardial dysfunction in HCM beyond conventional markers. Global LV torsion, in particular, emerges as a sensitive and robust non-invasive marker with diagnostic and prognostic potential.

## 1. Introduction

Hypertrophic cardiomyopathy (HCM) is the most common inherited cardiac disease, characterized by unexplained left ventricular (LV) hypertrophy, often with preserved ejection fraction and significant phenotypic heterogeneity. Although many patients remain asymptomatic, HCM is associated with an increased risk of sudden cardiac death, arrhythmias, and progressive heart failure [1,2]. Cardiac magnetic resonance (CMR) has become essential for diagnosis and risk stratification in HCM, particularly due to its accuracy in assessing wall thickness, ventricular volumes, and myocardial fibrosis via late gadolinium enhancement (LGE) [3,4].

In recent years, myocardial strain analysis using CMR feature-tracking (CMR-FT) has gained increasing attention as a sensitive marker of early systolic dysfunction. Parameters such as global longitudinal strain (GLS), global circumferential strain (GCS), and global radial strain (GRS) have shown prognostic relevance in various cardiovascular diseases, including ischemic cardiomyopathy, myocarditis, and heart failure with preserved ejection fraction [5,6]. However, in HCM, data on CMR-derived strain remain limited, and standardized strain profiles for this population have yet to be defined [7,8].

Preliminary evidence suggests that strain abnormalities in HCM—particularly reduced GLS—may correlate with the extent of myocardial fibrosis, hypertrophy severity, and arrhythmic burden, even in patients with preserved LVEF. Studies have also shown that impaired strain parameters may identify higher-risk individuals and precede structural remodeling, offering potential for earlier intervention and refined risk stratification [9]. Nonetheless, strain analysis in HCM remains underutilized, partly due to variability in acquisition and analysis methods, and the lack of disease-specific reference ranges.

Recent studies have reinforced the prognostic value of CMR-derived strain parameters in HCM. A 2023 multicenter study by Xu et al. demonstrated that reduced GLS and GCS were independently associated with adverse cardiovascular outcomes, including ventricular arrhythmias and heart failure hospitalizations, even in patients with preserved LVEF [10]. Importantly, regional strain abnormalities were found to precede LGE-detectable fibrosis, suggesting that strain imaging may capture early subclinical myocardial dysfunction before structural remodeling becomes evident.

Similarly, in a 2024 analysis, impaired GLS (≤15%) and GCS (≤18%) were shown to be strong predictors of major adverse cardiac events (MACE) in patients with both HCM and other non-ischemic cardiomyopathies. These findings underscore the incremental prognostic value of strain analysis over conventional metrics and support its integration into routine CMR protocols for risk stratification across a spectrum of cardiomyopathies [11].

Regarding LV torsion, it has emerged as a key marker of myocardial mechanics, offering deeper insight into both systolic and diastolic function beyond conventional measures. As interest in preclinical cardiac dysfunction grows, the assessment of LV torsion by cardiac magnetic resonance (CMR) provides a promising tool for detecting subtle alterations in myocardial performance [12]. However, data are scarce in patients with HCM. Zhong et al. recently showed that LV torsion was increased in HCM patients without LGE compared with both LGE-positive patients and healthy controls, and that reduced torsion was independently associated with the presence of myocardial fibrosis detected by LGE [13].

However, despite the growing use of CMR feature-tracking in myocardial disease, there is a lack of comprehensive studies integrating both strain and torsion analysis with tissue characterization parameters such as LGE, T1 mapping, and ECV in patients with HCM. In particular, the diagnostic and prognostic roles of LV torsion remain insufficiently explored, especially in individuals with preserved LVEF and variable fibrosis burden. This gap limits our ability to detect early myocardial dysfunction and to refine risk stratification beyond conventional imaging markers.

The aim of our study was to characterize left ventricular strain parameters—GLS, GCS, and GRS—using CMR-FT in patients with HCM and to explore their relationship with myocardial structure and clinical features. By identifying potential deformation patterns, we aim to clarify the role of strain imaging in the diagnostic and prognostic assessment of HCM.

## 2. Methods

### 2.1. Study Population

The study group consisted of 150 patients with confirmed HCM diagnosis, for which the HCM diagnosis was based on the current ESC guidelines, including the presence of unexplained left ventricular hypertrophy ≥ 15 mm in the absence of loading conditions sufficient to explain the degree of hypertrophy [14,15], who were evaluated between March 2019 and December 2024 at the 2nd Department of Internal Medicine, Iuliu Hațieganu University of Medicine and Pharmacy, Cluj-Napoca, Romania. CMR scans were per-formed at Affidea Hiperdia Diagnostic Imaging Center, Cluj-Napoca. Additionally, 100 matched healthy volunteers, free of overt cardiovascular illnesses, who underwent a simi-lar evaluation protocol, served as the control group. The evaluation protocol included medical history, physical examination, electrocardiography, transthoracic echocardiography, blood analysis, and CMR scanning. Exclusion criteria included significant coronary artery disease (defined as ≥50% luminal stenosis), moderate-to-severe valvular heart disease, history of cardiac surgery, contraindications to CMR (e.g., implanted non-MRI-compatible devices), and poor image quality precluding strain or tissue characterization analysis.

A priori sample size estimation was performed based on previously reported differences in GLS and LV torsion between HCM patients and healthy individuals. Assuming a medium effect size (Cohen’s d ≈ 0.6), 90% statistical power, and a two-sided alpha of 0.05, a minimum of 120 HCM patients and 80 controls were required. To enhance the robustness of subgroup analyses and multivariable modeling, we prospectively included 150 HCM patients and 100 controls. This approach ensured sufficient power to detect clinically relevant differences in CMR-derived parameters.

This study was approved by the Ethics Committee of Iuliu Hațieganu University of Medicine and Pharmacy (decision number 257/30 June 2021) and adhered to the Declaration of Helsinki. All participants provided written consent prior to inclusion.

### 2.2. CMR Scanning

CMR was performed using a Siemens 1.5 T Open Bore system (Magnetom Altea, Sie-mens Medical Solutions, Erlangen, Germany) using standardized international protocols [16,17]. Steady-state free precession (SSFP) cine sequences were acquired in standard short- and long-axis views. LGE sequences were assessed in all views 10 min after the intravenous administration of 0.2 mmol/kg gadoxetic acid (Clariscan, GE Healthcare, Oslo, Nor-way) using a segmented inversion–recovery gradient–echo sequence. Both native and post-contrast (minimum 15 min) T1-mapping sequences were evaluated in three short-axis views (basal, mid-ventricular, and apical). Extracellular volume fraction (ECV) was calculated using each patient’s hematocrit.

Cine-CMR-based volumetry and functional parameters were analyzed using Syngo Virtual Cockpit Version VB11A software [11]. All volumes were indexed to body surface area. LGE was assessed on short-axis images using a 17-segment model, with a threshold of 5 SD above the normal myocardial signal intensity. The LGE extent was reported in grams and as a percentage of LV mass.

**Figure 1:**
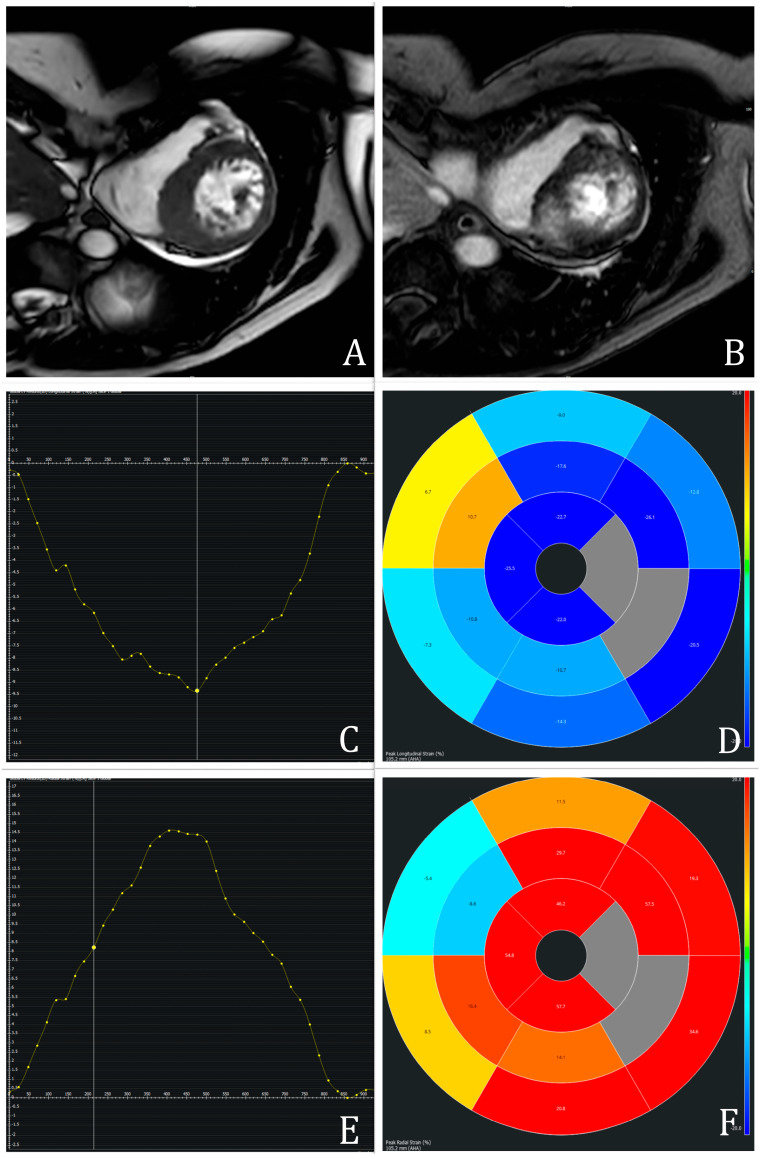
Example of CMR assessment in a patient with hypertrophic cardiomyopathy: Cine-short-axis view representing asymmetric septal hypertrophy of 21 mm (**A**) with corresponding midwall fibrosis determined with inversion–recovery late gadolinium enhancement image (**B**). Feature-Tracking-CMR with global longitudinal (**C**,**D**) and radial (**E**,**F**) strain parameters. The y-axis represents the percent of myocardial deformation, whereas the x-axis shows the corresponding time during the cardiac cycle.

LV biomechanics, including GLS, GCS, GRS, and peak systolic/diastolic torsion, were analyzed via feature tracking CMR. Post-processing was performed using cvi42 (Circle Cardiovascular Imaging, version 6.0.0, Calgary, AB, Canada) according to the published guidelines [7]. GLS and GRS were derived from two-, three-, and four-chamber long-axis views, while GCS was calculated from basal, mid, and apical short-axis views. Endocardial and epicardial contours were manually traced at end-diastole and end-systole, respectively. LV segmentation followed the American Heart Association 17-segment model, excluding segment 17 [12]. Structures such as LVOT, papillary muscles, trabeculae, pericardium, and epicardial fat were excluded. An example of CMR-FT measurements is represented in Figure 1.

LV torsion was calculated as the difference in rotational angle between apical and basal slices at peak systole, divided by the longitudinal distance and expressed in degrees per centimeter. Only segments with adequate tracking were included. Two independent observers performed the measurements; intra- and inter-observer variability was assessed on a random subset. This method has been validated against myocardial tagging and DENSE [18,19].

A total of 261 participants were initially enrolled. After image quality assessment, 11 individuals (7 with HCM and 4 controls) were excluded due to incomplete datasets or poor tracking quality. Thus, feature-tracking analysis was successfully performed in the final study cohort of 250 participants (150 HCM patients and 100 controls), yielding a success rate of 95.8%.

### 2.3. Reproducibility Analysis

To ensure the reliability of image-derived parameters, we performed a reproducibility analysis on a random subset of 20 patients representative of the study cohort. Both intra-observer and inter-observer variability were assessed for key functional and tissue metrics, including global longitudinal strain (GLS), global circumferential strain (GCS), global radial strain (GRS), and global LV torsion. The prerecorded data were used to determine specific parameters, which were assessed independently by two researchers, each performing two measurements per case. All observers were blinded to clinical and imaging results during evaluation.

Intraclass correlation coefficients (ICCs) were calculated using a two-way random-effects model with absolute agreement. Additionally, Kappa coefficients (K) were used to quantify reproducibility across measurements. The intra-observer reproducibility yielded K-values of 0.93 for GLS, 0.91 for GCS, and 0.92 for LV torsion. Inter-observer agreement was also strong, with K-values of 0.85 for GLS, 0.88 for GCS, and 0.84 for LV torsion. These results confirm a high level of consistency in both strain and torsion measurements.

Overall, all assessed parameters demonstrated excellent reproducibility (ICC > 0.90), supporting the robustness of CMR-FT–derived functional metrics. The strong agreement between observers ensures that measurement variability is unlikely to have significantly influenced the validity of our results.

### 2.4. Statistical Analysis

Continuous variables were expressed as the mean ± standard deviation (SD) or median with interquartile range (IQR), depending on their distribution, which was assessed using the Shapiro–Wilk test. Categorical variables were reported as absolute numbers and percentages. Comparisons between groups (HCM vs. controls) were performed using the unpaired Student’s *t*-test or the Mann–Whitney U test for continuous variables, and the chi-square or Fisher’s exact test for categorical variables, as appropriate. Correlations between myocardial strain parameters and CMR-derived structural markers (LGE extent, T1 values, ECV) were evaluated using Spearman’s rank correlation coefficient (r). A two-tailed *p*-value < 0.05 was considered statistically significant. ROC curve analysis was performed to evaluate the discriminatory ability of CMR-derived myocardial mechanics in distinguishing between patients with and without the target diagnosis. Statistical analyses were conducted using IBM SPSS Statistics version 27 (IBM Corp., Armonk, NY, USA).

## 3. Results

### 3.1. Baseline Characteristics 

This study included 150 patients with HCM and 100 healthy controls. The baseline characteristics are presented in Table 1. The two groups were similar in age (59 vs. 58 years, *p* = 0.620) and sex distribution (63% vs. 61% males, *p* = 0.710). HCM patients showed significantly higher body mass index (BMI) (30 vs. 29 kg/m^2^, *p* = 0.031), systolic blood pressure (130 vs. 109 mmHg, *p* < 0.001), and NTproBNP levels (1340 vs. 220 pg/mL, *p* < 0.001). The triglyceride levels were paradoxically lower in the HCM group (119 vs. 176 mg/dL, *p* < 0.001), while total cholesterol was similar. The proportion of active smokers was higher among HCM patients (35% vs. 32%, *p* = 0.032), and a significantly greater number were classified as NYHA class II or III at the time of CMR (61% vs. 15%, *p* < 0.001). Estimated GFR was slightly higher in the HCM group (87 vs. 82 mL/min/1.73 m^2^, *p* = 0.016)

### 3.2. CMR Characteristics

The standard CMR parameters are summarized in Table 2. HCM patients had significantly increased indexed LV end-diastolic (75 vs. 63 mL/m^2^) and end-systolic volumes (26 vs. 22 mL/m^2^), as well as a markedly elevated LV mass index (97 vs. 57 g/m^2^), all with *p* < 0.001. Despite this, LVEF was comparable between groups (65% vs. 66%, *p* = 0.290).

Extensive myocardial fibrosis was observed in HCM patients, with a median LGE mass of 33 g, representing 15% of total LVM. The dominant LGE pattern was nodular (57%), followed by linear (29%). Native T1 values (1011 vs. 972 ms) and ECV (27% vs. 26%) were significantly elevated in the HCM group (*p* < 0.001 for both), suggesting increased interstitial matrix remodeling. Left atrial volume was higher in HCM patients (78 vs. 53 mL, *p* < 0.001), with positive LA-LGE present in 41%. Right ventricular volumes were reduced in HCM, but RVEF was preserved and not significantly different between groups.

Among the 150 HCM patients, 121 (80.7%) were LGE-positive and 29 (19.3%) LGE-negative. Comparative analysis between these subgroups revealed no statistically significant differences in strain or torsion parameters, although LGE-negative patients showed a trend toward more preserved mechanics. Specifically, median global longitudinal strain (GLS) was slightly better in LGE-negative patients (−16.9% vs. −16.0%, *p* = 0.10), as was global circumferential strain (−18.5% vs. −17.6%, *p* = 0.09). Global LV torsion and its systolic and diastolic peaks were comparable between groups. Likewise, tissue characteristics such as native T1 and ECV showed no significant differences. These findings suggest that LGE-negative patients may exhibit marginally more favorable myocardial mechanics, though not reaching statistical significance in this cohort.

### 3.3. Biomechanical (Strain and Torsion) Parameters

All measured strain parameters were significantly impaired in HCM patients (Table 3). GLS was reduced to −16% (vs. −20% in controls), GCS to −18% (vs. −21%), and GRS to 29% (vs. 38%), all with *p* < 0.001. Global LV torsion was also markedly lower in HCM (1.27 vs. 1.95, *p* < 0.001), along with reduced peak-systolic (12°/cm vs. 13°/cm, *p* < 0.001) and peak-diastolic torsion (−10°/cm vs. −12°/cm, *p* < 0.001). These findings indicate widespread subclinical myocardial dysfunction, despite preserved LVEF.

### 3.4. Myocardial Strain Profiles in HCM vs. Controls

In the subgroup analysis, HCM patients (n = 52) exhibited significantly lower values across all myocardial deformation parameters compared with controls (n = 50). Mean GLS was markedly reduced in HCM patients (−15.4% vs. −19.8%), as were GCS (−17.1% vs. −21.3%) and GRS (28.2% vs. 38.9%). Similarly, global LV torsion was significantly diminished (1.18 vs. 1.96), indicating impaired systolic mechanics. Peak systolic torsion was lower in HCM (mean: 10.9°/cm vs. 12.9°/cm), while peak diastolic untwisting was also attenuated (−9.7°/cm vs. −11.2°/cm), suggesting delayed or incomplete mechanical relaxation.

These differences further support the presence of global myocardial dysfunction in HCM despite preserved LVEF. Additionally, the wide standard deviations in strain values among HCM patients suggest heterogeneous mechanical impairment, potentially reflecting underlying structural variability such as fibrosis or wall thickness distribution.

### 3.5. Associations Between Strain Metrics and Myocardial Tissue Characteristics

Spearman correlation analysis revealed significant associations between biomechanical strain parameters and CMR-derived structural markers in the HCM cohort. GLS was positively correlated with native T1-mapping (r = 0.481; *p* < 0.0001), ECV (r = 0.350; *p* < 0.0001), indexed LVM (r = 0.474; *p* < 0.0001), and peak-diastolic LV torsion (r = 0.367, *p* < 0.0001), and negatively correlated with global LV torsion (r = −0.633; *p* < 0.0001) and peak-systolic LV torsion (r = −0.547; *p* < 0.0001). GLS was moderately and negatively correlated with the extent of late gadolinium enhancement (LGE %) (r = −0.314, *p* < 0.001), suggesting that impaired longitudinal deformation reflects underlying myocardial fibrosis. Similar associations were observed for GCS (r = −0.331, *p* < 0.001), while GRS showed a positive correlation with LGE (r = 0.322, *p* < 0.001), likely reflecting compensatory radial thickening in non-fibrotic segments.

Global torsion did not significantly correlate with LGE extent (r = −0.008, *p* = NS), but showed weak associations with other functional parameters. No meaningful correlations were observed between strain parameters and LVEF or LV mass index, highlighting their independence from traditional systolic function markers.

**Figure 2:**
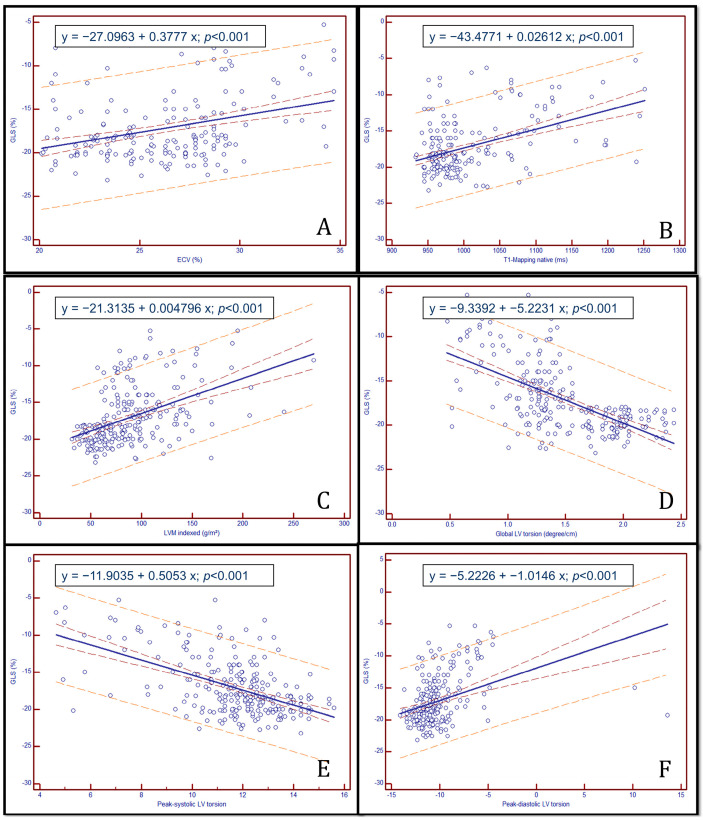
Correlation between global longitudinal strain and cardiac MRI-derived tissue and functional parameters in a patient cohort: GLS vs. (**A**) extracellular volume (ECV, %), (**B**) native T1 mapping (ms), (**C**) left ventricular mass index (LVMi, g/m), (**D**) global left ventricular torsion (°/cm), (**E**) peak diastolic LV torsion (°/cm), (**F**) peak systolic LV torsion (°/cm).

Scatter plots showing linear relationships between GLS and biomechanical strain parameters and CMR-derived structural markers in the HCM cohort are shown in Figure 2.

### 3.6. Receiver Operating Characteristic (ROC) Analysis

Among the evaluated CMR-derived parameters, global LV torsion showed the highest diagnostic performance, with an AUC of 0.995 (95% CI: 0.976–1.000; SE = 0.00473), reflecting near-perfect discrimination. This was followed by GLS, with an AUC of 0.877 (95% CI: 0.829–0.915; SE = 0.0225), and native T1 mapping, with an AUC of 0.731 (95% CI: 0.671–0.785; SE = 0.0311). The lowest performance was observed for ECV, which yielded an AUC of 0.657 (95% CI: 0.595–0.716; SE = 0.0339). 

**Figure 3:**
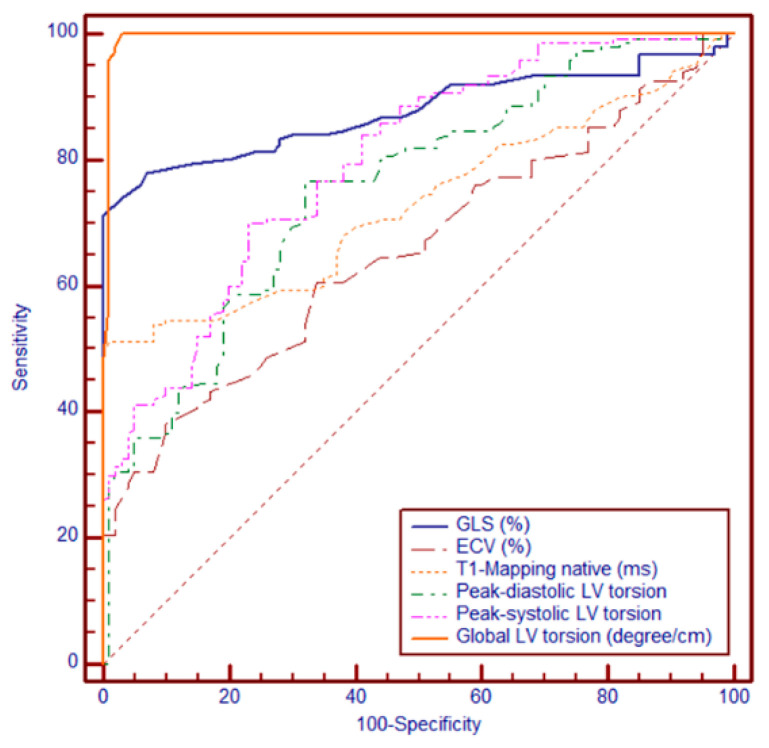
Left ventricular torsion below 0.7°/cm is associated with impaired prognosis in patients with hypertrophic cardiomyopathy.

Using Youden’s Index to identify the optimal cut-off, a threshold value of 0.7°/cm was determined as the most informative, providing the best balance between sensitivity and specificity. This cut-off was subsequently used for risk stratification in Kaplan–Meier survival analyses and subgroup comparisons. Global LV torsion significantly outperformed ECV, T1, and GLS (all *p* < 0.0001), GLS was superior to both T1 (*p* = 0.0001) and ECV (*p* < 0.0001), whereas T1 showed a modest but significant advantage over ECV (*p* = 0.0097). These findings highlight the incremental diagnostic value of global LV torsion, which surpassed both fibrosis-related markers and myocardial strain parameters in distinguishing between patient groups (Figure 3).

Kaplan–Meier survival curves comparing event-free survival in patients with hypertrophic cardiomyopathy (HCM) stratified by left ventricular (LV) torsion values were established. Patients with LV torsion < 0.7°/cm (red line) exhibited significantly lower event-free survival compared with those with torsion ≥ 0.7°/cm (blue line). At 24 months, approximately 70% of patients in the low-torsion group remained event-free, compared with over 90% in the preserved torsion group. This finding suggests that reduced LV torsion is a strong predictor of adverse clinical outcomes in HCM.

ECV = extracellular volume; GLS = global longitudinal strain; LV = left ventricle. Abbreviations: GLS = global longitudinal strain; ECV = extracellular volume; LV = left ventricle; °/cm = degrees per centimeter.

## 4. Discussion

In this study, we assessed LV deformation and torsion parameters using CMR-FT in patients with HCM. Our findings confirm that, despite preserved LVEF, patients with HCM exhibit significant impairment in GLS, GCS, GRS, and LV torsion, indicating subclinical myocardial dysfunction. Moreover, the ROC analysis demonstrated that global LV torsion provided significantly superior discriminatory power compared with GLS, native T1, and ECV, suggesting its potential as a robust diagnostic marker. These results reinforce existing evidence that conventional measures such as LVEF may underestimate early myocardial impairment in HCM [18,20].

Among strain parameters, GLS was the most consistently and significantly reduced, supporting its role as a sensitive biomarker of early systolic dysfunction. Prior studies have similarly shown that impaired GLS is predictive of ventricular arrhythmias, HF symptoms, and SCD in HCM [9,21]. Our findings also revealed moderate negative correlations between GLS and GCS with LGE extent, suggesting that strain alterations reflect underlying fibrotic burden [20]. Interestingly, we identified significantly impaired GLS and GCS, even in patients with minimal or absent LGE. This supports the hypothesis that strain abnormalities may precede detectable structural remodeling on CMR, and therefore hold diagnostic value in earlier disease stages [3,5,22]. These findings are in line with those of previous work demonstrating subclinical dysfunction in mutation carriers or genotype-positive/phenotype-negative individuals [10].

In our study, LV torsion emerged as a highly sensitive marker of myocardial dysfunction. Compared with healthy controls, HCM patients demonstrated significantly reduced global LV torsion, as well as lower peak systolic and diastolic torsion values, indicating impaired twist–untwist mechanics and subtle alterations in both systolic and diastolic function. These biomechanical changes were not reflected in conventional parameters such as LVEF, underlining the added value of torsion analysis. Interestingly, global LV torsion did not correlate significantly with the extent of myocardial fibrosis as quantified by LGE, but showed moderate associations with strain parameters like GLS and GCS, suggesting a more complex interplay between myocardial mechanics and structural remodeling. This aspect is, to some extent, similar to the findings of Zhong et al. [13]. Moreover, ROC analysis further highlighted the diagnostic strength of global LV torsion, which achieved an AUC of 0.995—surpassing that of all other CMR-derived markers including GLS, native T1, and ECV. These findings support the use of CMR-derived torsion as a powerful, non-invasive biomarker for early functional impairment in HCM, with potential clinical relevance for risk stratification and disease monitoring.

However, other studies report only weak-to-moderate correlations between CMR-derived strain and myocardial tissue characteristics, suggesting that these measurements may capture different pathophysiological processes [11,19]. In particular, GRS appears to be less specific, and its interpretation may be confounded by compensatory hypertrophy or regional loading conditions. Furthermore, strain parameters did not correlate with LVM or LVEF in our cohort, highlighting their independence from conventional metrics. 

Interestingly, although GRS was significantly reduced in HCM patients compared with controls, its relative preservation compared with GLS and GCS may reflect a compensatory mechanism. In hypertrophied myocardium, particularly in regions not extensively affected by fibrosis, radial thickening may be augmented to preserve stroke volume despite impaired longitudinal and circumferential mechanics. This phenomenon could be partially explained by the altered myocardial fiber architecture in HCM, where mid-wall circumferential and oblique fibers contribute more actively to radial displacement. Furthermore, heterogeneous patterns of patchy fibrosis, often localized to specific segments, may spare adjacent areas that hyperfunction radially in response to increased regional wall stress. These dynamics underscore the complex interplay between structural remodeling and biomechanical adaptation in HCM.

Methodological variability remains a major limitation in widespread adoption of strain analysis. Differences in vendor software, segmentation technique, and frame rate significantly influence results [20]. While our study used a standardized protocol, inter-study comparability is still challenging, and normative data for HCM populations remain insufficiently established [14].

From a prognostic standpoint, several reports now support the additive value of GLS and GCS over LGE in predicting MACE in HCM, particularly when values drop below −15% and −18%, respectively [11,23]. These strain thresholds may refine current risk stratification models and identify patients at higher risk who may otherwise appear low-risk by LGE or ESC HCM Risk-SCD scores. Technological advances, such as the implementation of deep learning algorithms in CMR-FT workflows, have shown promise in improving measurement reproducibility [24]. As CMR-FT matures and standardized cutoffs are validated, integration of strain into clinical workflows will become more feasible, especially in specialized cardiomyopathy centers.

While our findings suggest that strain and torsion may offer incremental value beyond conventional risk factors, especially in identifying subclinical dysfunction, their integration into established risk stratification tools—such as the ESC HCM Risk-SCD model—remains hypothetical. The present analysis was cross-sectional and not designed to assess clinical outcomes. Therefore, although the observed associations are biologically plausible and potentially meaningful, prospective longitudinal studies with hard endpoints (e.g., arrhythmias, SCD, and ICD interventions) are required before these parameters can be formally incorporated into clinical algorithms. Until such data are available, the role of myocardial mechanics in risk stratification should be interpreted with caution.

In conclusion, our findings emphasize the diagnostic and prognostic value of CMR-derived strain analysis in HCM. GLS and GCS emerge as robust markers of subclinical dysfunction, with potential to improve early detection and risk assessment beyond conventional imaging markers. Future multicenter studies with long-term follow-up are needed to further define their role in guiding therapeutic strategies, particularly in phenotype-negative or intermediate-risk patients.

## 5. Study Limitations

This study has several limitations. First, its single-center, cross-sectional design limits causal inference and precludes the assessment of the predictive value of strain parameters over time. As a single-center study conducted using uniform imaging protocols and a relatively homogeneous regional population, the generalizability of these results to other clinical settings or ethnically diverse cohorts may be limited. Second, although CMR was performed using a standardized protocol, feature-tracking analysis remains operator-dependent and may vary across platforms. Third, LGE quantification, while standardized, is less sensitive than T1 mapping in detecting diffuse fibrosis. The modest number of LGE-negative patients may also reduce the statistical power of subgroup comparisons. Additionally, the relatively homogenous ethnic background of the cohort may limit generalizability, and future multi-ethnic studies are needed to validate these findings. The absence of genetic data further restricts interpretation, as sarcomeric mutations can influence myocardial mechanics even before overt hypertrophy. Last, but not least, without genotyping, important mechanistic subgroups may remain unrecognized.

## 6. Conclusions

Our findings demonstrate that CMR-derived strain parameters—particularly GLS and GCS—are significantly impaired in patients with HCM, even in the presence of pre-served ejection fraction and in the absence of detectable LGE. These abnormalities correlate with myocardial fibrosis but may also reflect early subclinical dysfunction in patients without overt structural remodeling. Strain analysis offers independent and complementary insights beyond traditional imaging markers and may help to refine risk stratification in HCM. Future prospective studies are warranted to validate the prognostic value of strain parameters and their integration into multimodal risk assessment models for clinical decision-making.

## Figures and Tables

**Table 1:** Baseline characteristics.

Variable	HCM Group (n = 150)	Control Group (n = 100)	*p*-Value	Statistical Test
Age, years	59 (50 to 65)	58 (49 to 65)	0.620	U = 7234
Males, n (%)	95 (63%)	61 (61%)	0.710	χ^2^ = 0.14
BMI, kg/m^2^	30 (27 to 32)	29 (26 to 31)	0.031	U = 6021
Hypercholesterolemia, mg/dL	203 (163 to 235)	197 (177 to 234)	0.671	U = 7260
Triglycerides, mg/dL	119 (95 to 179)	176 (143 to 212)	<0.001	U = 4320
eGFR, ml/min/1.73 m^2^	87 (73 to 99)	82 (70 to 93)	0.016	U = 6132
Systolic BP, mmHg	130 (120 to 150)	109 (105 to 114)	<0.001	U = 3101
Diabetes mellitus type 2, n (%)	40 (27%)	29 (29%)	0.687	χ^2^ = 0.16
Smokers, n (%)	53 (35%)	32 (32%)	0.032	χ^2^ = 4.59
NYHA class at time of CMR, n (%)	I: 26 (17%)	I: 15 (15%)	<0.001	χ^2^ = 36.9
	II: 68 (45%)			
	III: 24 (16%)			
L	1340 (745 to 2600)	220 (130 to 300)	<0.001	U = 2920
Global LV torsion, °/cm	−2.1 (−2.5 to −1.7)	−2.7 (−3.1 to −2.2)	<0.001	U = 3280
Peak systolic LV torsion, °/cm	−2.5 (−3.1 to −2.0)	−3.2 (−3.6 to −2.8)	<0.001	U = 3100
Peak diastolic LV torsion, °/cm	1.6 (1.2 to 2.0)	2.0 (1.7 to 2.3)	0.002	U = 4301

Continuous variables are presented as the median (interquartile range); categorical variables as n (%). Statistical comparisons were performed using the Mann–Whitney U test for continuous variables and the Chi-square test for categorical variables. Abbreviations: BMI, body mass index; CMR, cardiac magnetic resonance; eGFR, estimated glomerular filtration rate; HCM, hypertrophic cardiomyopathy; NTproBNP, N-terminal prohormone of brain natriuretic peptide; NYHA, New York Heart Association; BP, blood pressure; LV, left ventricle; °/cm, degrees per centimeter.

**Table 2:** CMR characteristics of the studied population.

Variable	HCM Group (n = 150)	Control Group (n = 100)	*p*-Value	Test Statistic
LVEDV indexed, mL/m^2^	75 (65 to 92)	63 (51 to 76)	<0.001	U = 2985
LVESV indexed, mL/m^2^	26 (20 to 35)	22 (16 to 26)	<0.001	U = 3150
LVM indexed, g/m^2^	97 (83 to 122)	57 (48 to 68)	<0.001	U = 2700
LVEF, %	65 (60 to 69)	66 (62 to 68)	0.290	U = 6752
LGE mass, g	33 (12 to 48)	-/-	-	-
LGE mass/LVM, %	15 (7 to 25)	-/-	-	-
LGE pattern—nodular, n (%)	86 (57%)	-/-	-	-
LGE pattern—linear, n (%)	43 (29%)	-/-	-	-
LGE pattern—focal, n (%)	3 (2%)	-/-	-	-
T1-Mapping native, ms	1011 (968 to 1081)	972 (959 to 987)	<0.001	U = 3400
ECV, %	27 (25 to 29)	26 (23 to 28)	<0.001	U = 3980
RVEDV indexed, mL/m^2^	45 (34 to 56)	55 (47 to 66)	<0.001	U = 4100
RVESV indexed, mL/m^2^	17 (12 to 22)	41 (34 to 53)	<0.001	U = 3800
RVEF, %	62 (58 to 66)	62 (58 to 67)	0.947	U = 7445
Max. LA volume, mL	78 (71 to 87)	53 (48 to 57)	<0.001	U = 2600
Positive LA-LGE, n (%)	62 (41%)	-/-	-	-
Mitral regurgitation grade 1, n (%)	32 (21%)	-/-	-	-
Mitral regurgitation grade 2, n (%)	76 (51%)	-/-	-	-
Mitral regurgitation grade 3, n (%)	42 (28%)	-/-	-	-

Continuous variables are presented as the median (interquartile range); categorical variables as n (%). Statistical comparisons were performed using the Mann–Whitney U test for continuous variables and the Chi-square test for categorical variables. The symbol “-/-” indicates that the variable was not applicable or not assessed in the control group. Abbreviations: CMR, cardiac magnetic resonance; HCM, hypertrophic cardiomyopathy; LA, left atrium; LGE, late gadolinium enhancement; LVEDV, left ventricular end-diastolic volume; LVEF, left ventricular ejection fraction; LVESV, left ventricular end-systolic volume; LVM, left ventricular mass; RVEDV, right ventricular end-diastolic volume; RVEF, right ventricular ejection fraction; RVESV, right ventricular end-systolic volume.

**Table 3:** Comparison of CMR-FT-determined strain and torsion parameters between HCM patients and healthy controls.

Variable	HCM Group (n = 150)	Control Group (n = 100)	*p*-Value	Test Statistic
GLS, %	−16 (−18 to −13)	−20 (−20 to −19)	<0.001	U = 2500
GCS, %	−18 (−20 to −14)	−21 (−22 to −20)	<0.001	U = 2600
GRS, %	29 (25 to 32)	38 (33 to 43)	<0.001	U = 2750
Global LV-torsion, °/cm	1.27 (1.09 to 1.36)	1.95 (1.82 to 2.05)	<0.001	U = 2900
Peak-systolic LV-torsion, °/cm	12 (10 to 12)	13 (12 to 14)	<0.001	U = 3000
Peak-diastolic LV-torsion, °/cm	−10 (−11 to −9)	−12 (−12 to −11)	<0.001	U = 3100

Continuous variables are presented as median (interquartile range). Statistical comparisons were performed using the Mann–Whitney U test for all variables. Abbreviations: CMR, cardiac magnetic resonance; GCS, global circumferential strain; GLS, global longitudinal strain; GRS, global radial strain; HCM, hypertrophic cardiomyopathy; LV, left ventricle.

## Data Availability

The original contributions presented in this study are included in the article. Further inquiries can be directed to the corresponding author(s).
